# Supplementary material for: Capillary pericytes express α-smooth muscle actin, which requires prevention of filamentous-actin depolymerization for detection
Source: eLife. 2018 Mar 21;7:e34861. doi: 10.7554/eLife.34861 (PMC5862523; doi:10.7554/eLife.34861)
Supplement: Supplementary file 1. — The table summarizes all experiments performed including the treatment groups and number of mice used. Where appropriate, analyses, statistical comparisons, mean ±SEM, and P values are also indicated. Please note that some animals/retinas were used for more than one experiment, therefore, the total number of mice is less than total number of experiments. [file elife-34861-supp1.docx]

| **Experiment** | | **Group** | | **n of mice** | | |  |
| --- | --- | --- | --- | --- | --- | --- | --- |
| Fixation method | | Antigen retrieval | | 2 | | |  |
|  |  | Ethanol | | 4 | | |  |
|  |  | Methanol + non-fluorescent phalloidin | | 4 | | |  |
|  |  | Methanol + NG2-DsRed or PDGFRb colocalization | | 8 | | |  |
|  |  | Jasplakinolide | | 3 | | |  |
|  |  | γ-actin colocalization/labeling | | 6 | | |  |
|  |  | PFA + F-actin labeling | | 3 | | |  |
|  |  | Negative control | | 3 | | |  |
| siRNA experiments | | Transfection | | **11** | | |  |
| **Experiment** | **Analysis** | **Comparison** | **Number of SMA+ vessels (mean±SEM)** | **n of mice** | **P** | |  |
| **Fixation methods** | **Total number of vessels** | **PFA vs. methanol** | 254 ± 63 vs. 441 ± 28 | 6 vs. 5 | 0.023* (ANOVA) | |  |
|  |  | **PFA vs. methanol phalloidin** | 254 ± 63 vs. 448 ± 57 | 6 vs. 5 | 0.023*  (ANOVA) | |  |
|  |  | **Methanol vs. methanol phalloidin** | 441 ± 28 vs. 448 ± 57 | 5 vs. 5 | n.s.  (ANOVA) | |  |
|  | **Number of vessels (order 1)** | **PFA vs. methanol** | 7 ± 0.4 vs. 7 ± 0.6 | 6 vs. 5 | n.s.  (ANOVA) | |  |
|  |  | **PFA vs. methanol phalloidin** | 7 ± 0.4 vs. 7 ± 0.4 | 6 vs. 5 | n.s.  (ANOVA) | |  |
|  |  | **PFA vs. methanol jasplakinolide** | 7 ± 0.4 vs. 7 ± 0.7 | 5 vs. 3 | n.s.  (ANOVA) | |  |
|  | **Number of vessels (order 2)** | **PFA vs. methanol** | 60 ± 5.6 vs. 63 ± 10.2 | 6 vs. 5 | n.s.  (ANOVA) | |  |
|  |  | **PFA vs. methanol phalloidin** | 60 ± 5.6 vs. 51 ± 10.8 | 6 vs. 5 | n.s.  (ANOVA) | |  |
|  |  | **PFA vs. methanol jasplakinolide** | 60 ± 5.6 vs. 37 ± 6.1 | 5 vs. 3 | n.s.  (ANOVA) | |  |
|  | **Number of vessels (order 3)** | **PFA vs. methanol** | 113 ± 24.3 vs. 173 ± 16.1 | 6 vs. 5 | n.s.  (ANOVA) | |  |
|  |  | **PFA vs. methanol phalloidin** | 113 ± 24.3 vs. 129 ± 20.4 | 6 vs. 5 | n.s.  (ANOVA) | |  |
|  |  | **PFA vs. methanol jasplakinolide** | 113 ± 24.3 vs. 133 ± 22.6 | 5 vs. 3 | n.s.  (ANOVA) | |  |
|  | **Number of vessels (order 4)** | **PFA vs. methanol** | 60 ± 28.0 vs. 144 ± 19.1 | 6 vs. 5 | 0.003* (ANOVA) | |  |
|  |  | **PFA vs. methanol phalloidin** | 60 ± 28.0 vs. 139 ± 16.8 | 6 vs. 5 | n.s.  (ANOVA) | |  |
|  |  | **PFA vs. methanol jasplakinolide** | 60 ± 28.0 vs. 233 ± 29.0 | 5 vs. 3 | 0.003* (ANOVA) | |  |
|  | **Number of vessels (order 5)** | **PFA vs. methanol** | 15 ± 6.8 vs. 51 ± 12.2 | 6 vs. 5 | n.s.  (ANOVA) | |  |
|  |  | **PFA vs. methanol phalloidin** | 15 ± 6.8 vs. 80 ± 19.3 | 6 vs. 5 | <0.0001*  (ANOVA) | |  |
|  |  | **PFA vs. methanol jasplakinolide** | 15 ± 6.8 vs. 225 ± 27.6 | 5 vs. 3 | <0.0001*  (ANOVA) | |  |
|  | **Number of vessels (order 6)** | **PFA vs. methanol** | 0 ± 0.0 vs. 5 ± 4.8 | 6 vs. 5 | n.s.  (ANOVA) | |  |
|  |  | **PFA vs. methanol phalloidin** | 0 ± 0.0 vs. 23 ± 8.6 | 6 vs. 5 | n.s.  (ANOVA) | |  |
|  |  | **PFA vs. methanol jasplakinolide** | 0 ± 0.0 vs. 123 ± 30.2 | 5 vs. 3 | <0.0001*  (ANOVA) | |  |
|  | **Number of vessels (order ≥ 7)** | **PFA vs. methanol** | 0 ± 0.0 vs. 0 ± 0.0 | 6 vs. 5 | n.s.  (ANOVA) | |  |
|  |  | **PFA vs. methanol phalloidin** | 0 ± 0.0 vs. 20 ± 6.1 | 6 vs. 5 | 0.002*  (ANOVA) | |  |
|  |  | **PFA vs. methanol jasplakinolide** | 0 ± 0.0 vs. 31 ± 12.2 | 5 vs. 3 | 0.002*  (ANOVA) | |  |
|  | **Number of α-SMA+/NG2+ pericytes** | **PFA vs. methanol** | 509 ± 30.5 vs. 883 ± 56.1 | 3 vs. 3 | 0.035*  (ANOVA) | |  |
|  |  | **PFA vs. methanol phalloidin** | 509 ± 30.5 vs. 890 ± 138.2 | 3 vs. 3 | 0.035*  (ANOVA) | |  |
|  |  | **Methanol vs. methanol phalloidin** | 883 ± 56.1 vs. 890 ± 138.2 | 3 vs. 3 | n.s.  (ANOVA) | |  |
|  | **Number of α-SMA+ pericytes in superficial plexus** | **PFA vs. methanol** | 1196±255.1 vs. 1343±50.0 | 3 vs. 3 | n.s.  (ANOVA) | |  |
|  |  | **PFA vs. methanol phalloidin** | 1196 ± 255.1 vs.1388 ± 64.2 | 3 vs. 3 | n.s.  (ANOVA) | |  |
|  |  | **PFA vs. methanol jasplakinolide** | 1196 ± 255.1 vs.. 1492 ± 199.0 | 3 vs. 3 | n.s.  (ANOVA) | |  |
|  | **Number of α-SMA+ pericytes in intermediate plexus** | **PFA vs. methanol** | 97 ± 22.7 vs. 424 ± 71.6 | 3 vs. 3 | 0.034*  (ANOVA) | |  |
|  |  | **PFA vs. methanol phalloidin** | 97 ± 22.7 vs. 509 ± 78.5 | 3 vs. 3 | 0.011*  (ANOVA) | |  |
|  |  | **PFA vs. methanol jasplakinolide** | 97 ± 22.7 vs. 497 ± 99.5 | 3 vs. 3 | 0.012*  (ANOVA) | |  |
|  | **Number of α-SMA+ pericytes in deeper plexus** | **PFA vs. methanol** | 4 ± 2.7 vs. 119 ± 27.2 | 3 vs. 3 | n.s.  (ANOVA) | |  |
|  |  | **PFA vs. methanol phalloidin** | 4 ± 2.7 vs. 260 ± 29.8 | 3 vs. 3 | 0.021*  (ANOVA) | |  |
|  |  | **PFA vs. methanol jasplakinolide** | 4 ± 2.7 vs. 359 ± 95.7. | 3 vs. 3 | 0.003*  (ANOVA) | |  |
| **Experiment** | **Analysis** | **Cohorts** | **% of α-SMA+ pericytes compared to NG2+ pericytes** | **n of mice** | | |  |
| **Fixation methods** | **% of α-SMA+ pericytes for each plexus compared to NG2+ pericytes** | **PFA (superficial, intermediate, deeper plexus)** | **75.7, 10.4, and 0.4** | **3** | | |  |
|  |  | **Methanol (superficial, intermediate, deeper plexus)** | **85.1, 45.8, and 12.5** | **3** | | |  |
|  |  | **Phalloidin (superficial, intermediate, deeper plexus)** | **87.9, 54.9, and 27.3** | **3** | | |  |
|  |  | **Jasplakinolide (superficial, intermediate, deeper plexus)** | **78.7, 60.4, and 55.0** | **3** | | |  |
| **Experiment** | **Analysis** | **Comparison** | **% of continuous SMA+ vessels (mean±SEM)** | **n of mice** | | **P** |  |
| **% of continuous**  **α-SMA+ vessels after knocking down**  **α-SMA** | **α-SMA-siRNA-treated retinas** | **Order 1** | 66.67±33.33 | 3 | | 0.005  (non-parametric Jonckheere-terpstra test for trend analysis) |  |
|  |  | **Order 2** | 39.00±14.73 | 3 | |  |  |
|  |  | **Order 3** | 41.67±16.60 | 3 | |  |  |
|  |  | **Order 4** | 59.00±24.91 | 3 | |  |  |
|  |  | **Order 5** | 27.33±9.60 | 3 | |  |  |
|  |  | **Order 6** | 14.67±14.67 | 3 | |  |  |
|  |  | **Order 7** | **0**±0 | 3 | |  |  |
| **Experiment** | **Analysis** | **Comparison** | **Relative value** | **n of mice** | **P** | |  |
| **Amount of α-SMA after knocking down α-SMA** | **Western blot** | **Scrambled (control) siRNA-treated retinas vs. α-SMA-siRNA-treated retinas** | 1.22± 0.32 vs. 0.93 ± 0.23 | 3 vs. 3 | 0.4  (Two tailed Student’s *t*-test ) | |  |
|  | **qRT-PCR** | **Scrambled (control) siRNA-treated retinas vs. α-SMA-siRNA-treated retinas** | 1.51 ± 0.33vs. 1.14 ± 0.32 | 3 vs. 3 | 0.7  (Two tailed Student’s *t*-test ) | |  |
|  |  |  |  |  |  | |  |

**Supplementary file 1. Summary of experiments and comparisons.** The table summarizes all experiments performed including the treatment groups and number of mice used. Where appropriate, analyses, statistical comparisons, mean ± SEM, and P values are also indicated. Please note that some animals/retinas were used for more than one experiment, therefore, the total number of mice is less than total number of experiments.
